# Cost-utility of an eHealth application ‘Oncokompas’ that supports cancer survivors in self-management: results of a randomised controlled trial

**DOI:** 10.1007/s11764-020-00912-9

**Published:** 2020-07-12

**Authors:** A. van der Hout, F. Jansen, C. F. van Uden-Kraan, V. M. Coupé, K. Holtmaat, G. A. Nieuwenhuijzen, J. A. Hardillo, R. J. Baatenburg de Jong, N. L. Tiren-Verbeet, D. W. Sommeijer, K. de Heer, C. G. Schaar, R. J. E. Sedee, K. Bosscha, M. W. M. van den Brekel, J. F. Petersen, M. Westerman, J. Honings, R. P. Takes, I. Houtenbos, W. T. van den Broek, R. de Bree, P. Jansen, S. E. J. Eerenstein, C. R. Leemans, J. M. Zijlstra, P. Cuijpers, L. V. van de Poll-Franse, I. M. Verdonck-de Leeuw

**Affiliations:** 1 Department of Clinical, Neuro- and Developmental Psychology, Amsterdam Public Health Research Institute, Vrije Universiteit Amsterdam, Van der Boechorststraat 7, 1081 BT Amsterdam, The Netherlands; 2 Cancer Center Amsterdam (CCA), Amsterdam UMC, Amsterdam, The Netherlands; 3 Department of Otolaryngology – Head and Neck Surgery, Amsterdam UMC, Vrije Universiteit Amsterdam, Amsterdam, The Netherlands; 4 Department of Epidemiology and Biostatistics, Amsterdam UMC, Vrije Universiteit Amsterdam, Amsterdam, The Netherlands; 5 Department of Surgery, Catharina Hospital, Eindhoven, The Netherlands; 6 Department of Otolaryngology and Head and Neck Surgery, ErasmusMC Cancer Centre, Erasmus Medical Center, Rotterdam, The Netherlands; 7 Department of Hematology, Erasmus Medical Center, Rotterdam, The Netherlands; 8 Department of Internal Medicine, Flevoziekenhuis, Almere, The Netherlands; 9 Department of Medical Oncology, Amsterdam UMC, University of Amsterdam, Amsterdam, The Netherlands; 10 Department of Hematology, Amsterdam UMC, University of Amsterdam, Amsterdam, The Netherlands; 11 Department of Internal Medicine, Gelre ziekenhuis, Apeldoorn, The Netherlands; 12 Department of Otolaryngology, Head and Neck Surgery, Haaglanden MC, The Hague, The Netherlands; 13 Department of Surgery, Jeroen Bosch Ziekenhuis, Den Bosch, The Netherlands; 14 Department of Head and Neck Oncology and Surgery, Netherlands Cancer Institute, Amsterdam, The Netherlands; 15 Department of Hematology, Northwest Clinics, Alkmaar, The Netherlands; 16 Department of Otorhinolaryngology – Head and Neck Surgery, Radboud University Medical Center, Nijmegen, The Netherlands; 17 Department of Hematology, Spaarne Gasthuis, Hoofddorp, The Netherlands; 18 Department of Surgery, St. Anna Hospital, Geldrop, The Netherlands; 19 Department of Head and Neck Surgical Oncology, Utrecht University Medical Center, Utrecht, The Netherlands; 20 Department of Surgery, Elisabeth-TweeSteden Hospital, Tilburg, The Netherlands; 21 Department of Hematology, Amsterdam UMC, Vrije Universiteit Amsterdam, Amsterdam, The Netherlands; 22 Department of Research, Netherlands Comprehensive Cancer Organisation, Eindhoven, The Netherlands; 23 Division of Psychosocial Research & Epidemiology, Netherlands Cancer Institute, Amsterdam, The Netherlands; 24 CoRPS - Center of Research on Psychological and Somatic disorders, Department of Medical and Clinical Psychology, Tilburg University, Tilburg, The Netherlands

**Keywords:** Cancer survivorship, Supportive care, Self-management, Quality of life, eHealth, Cost-utility

## Abstract

**Purpose:**

The eHealth self-management application ‘Oncokompas’ was developed to support cancer survivors in monitoring health-related quality of life (HRQOL) and symptoms, and obtaining personalized feedback and options for supportive care. The aim of this study was to assess the cost-utility of Oncokompas compared with care as usual (CAU) among cancer survivors.

**Methods:**

Survivors were randomly allocated to the intervention or control group. Direct (non-)medical, indirect non-medical costs, and HRQOL were measured at 3- and 6-month follow-up, using iMTA Medical Consumption and Productivity Costs and the EuroQol-5D questionnaires. Mean cumulative costs and quality-adjusted life-years (QALYs) were compared between both groups.

**Results:**

In total, 625 survivors were randomized into intervention (*n* = 320) or control group (*n* = 305). Base case analysis showed that incremental costs from a societal perspective were − €163 (95% CI, − 665 to 326), and incremental QALYs were 0.0017 (95% CI, − 0.0121 to 0.0155) in the intervention group compared with those in the control group. The probability that, compared with CAU, Oncokompas is more effective was 60%, less costly 73%, and both more effective and less costly 47%. Sensitivity analyses showed that incremental costs vary between − €40 and €69, and incremental QALYs vary between − 0.0023 and − 0.0057.

**Conclusion:**

Oncokompas is likely to be equally effective on utilities, and not more expensive than CAU, and will therefore contribute to sustainable cancer survivorship care in a (cost-)effective manner.

**Implications for Cancer Survivors:**

Oncokompas seems to improve HRQOL and reduces the burden of several tumour-specific symptoms, while costs from a societal perspective are similar to CAU.

**Electronic supplementary material:**

The online version of this article (10.1007/s11764-020-00912-9) contains supplementary material, which is available to authorized users.

## Introduction

Cancer survivorship care includes physical rehabilitation, psychosocial care, lifestyle interventions, existential issues, and the (self-)management of survivors’ health and healthcare. It is, however, challenging to organise cancer survivorship care, because it is difficult to align individual needs and preferences with existing care options, and also to make cancer survivorship care available at acceptable costs [1, 2].

Data from patient-reported outcome measures (PROMs) can be used for optimal referral to supportive care in clinical practice. Behavioural intervention technologies (BITs) are currently often used to collect and process PROM data [3]. Also, eHealth self-management applications can support cancer survivors to self-manage their symptoms and health-related quality of life (HRQOL) [4–8]. However, not much is known yet on the cost-effectiveness or cost-utility of eHealth self-management applications and BITs among cancer survivors.

We developed Oncokompas, a fully automated BIT that supports cancer survivors by monitoring their HRQOL and symptoms; obtaining tailored feedback and advice on their physical, psychological and social functioning, lifestyle, and existential questions; and receiving a personalized overview of recommended supportive care services [9–13]. Oncokompas can be used by cancer survivors independently from a healthcare provider and follows a tailored care approach: all cancer survivors receive tailored information, advice, and tips; survivors with minor symptoms are referred to self-help interventions while survivors with major symptoms are primarily referred to professional care. Recently, we showed that Oncokompas has small, but significant effects on improving HRQOL and reducing the burden of several tumour-specific symptoms among cancer survivors [14].

During studies on the feasibility and implementation of Oncokompas [10, 13, 15], we observed that an important barrier among healthcare professionals and healthcare insurance companies to adopt and implement eHealth applications like Oncokompas was related to the uncertainty about costs and reimbursement. Also, it is important to know whether these applications may have a positive influence on costs from a societal perspective, including for example costs of absence from work, and costs of informal care.

The aim of the present study was to evaluate the cost-utility of Oncokompas compared with care as usual (CAU) among cancer survivors, from a societal and healthcare perspective.

## Methods

### Study design and participants

A randomised controlled trial was carried out among survivors of head and neck cancer, colorectal cancer, breast cancer, and lymphoma (including high- and low-grade non-Hodgkin lymphoma and Hodgkin lymphoma). These tumour types were chosen to ensure variability regarding age, sex, prevalent and less prevalent tumour types, solid and non-solid tumour types, cancer- and treatment-related symptoms, and the need for various types of supportive care. Other inclusion criteria were the following: age ≥ 18 years (no upper limit) and 3 months to 5 years after treatment with curative intent (all treatment modalities). Survivors who were still receiving endocrine therapy or immunotherapy, or had a wait-and-see regimen, were included 3 months after their previous treatment or diagnosis, respectively. Exclusion criteria were the following: no access to the Internet or no email address, severe cognitive impairment, insufficient mastery of the Dutch language, physical inability to complete a questionnaire, and male breast cancer survivors [14, 16].

The study protocol was approved by the Medical Ethics Committee of the VU University Medical Center (2015.523), published previously [16], and registered in the Netherlands Trial Register (NTR5774). All participants provided (online) written informed consent.

### Randomisation and masking

Cancer survivors who gave their informed consent were randomly allocated to the intervention group (direct access to Oncokompas) or CAU wait-list control group (access to Oncokompas after 6 months), in a 1:1 ratio. Randomisation was stratified by tumour type, and blocks with a length of 68 were used. Due to the nature of the intervention, participants could not be blinded for the allocated arm.

### Procedures

Participants were recruited through The Netherlands Cancer Registry (NCR) in 14 hospitals across The Netherlands, and invited by their (former) treating physician. Data collection was performed using the Patient-Reported Outcomes Following Initial Treatment and Long term Evaluation of Survivorship (PROFILES) registry [17].

### Intervention

Oncokompas supports cancer survivors in self-management, by monitoring (cancer-generic and tumour-specific) symptoms and HRQOL, providing feedback and information on their scores and a personalized overview of supportive care options, with the aim to reduce symptom burden and improve HRQOL [10, 12, 16]. In three steps, (1) Measure, (2) Learn, and (3) Act, users are guided through the application. In the component ‘Measure’, patient-reported outcome measures (PROMs) are completed on several domains, in ‘Learn’, data from the PROMs are processed in real-time, and with algorithms linked to tailored information and advice. In ‘Act’, an overview of healthcare options is given, based on PROMs, survivors’ expressed preferences, and the severity of symptoms. In case of elevated well-being risks, self-help options are offered, and in case of seriously elevated well-being risks, professional health-care options are offered. Oncokompas comprises generic modules for all cancer survivors, targeting physical, psychological and social functioning, lifestyle, and existential questions. Furthermore, tumour-specific modules are available for cancer survivors diagnosed with head and neck cancer, colorectal cancer, breast cancer, and (non-)Hodgkin lymphoma, covering problems related to the specific type of cancer. A cancer survivor can choose which topics he or she wants to address. Oncokompas was developed according to a participatory design approach, including all relevant stakeholders in each step of the development [9, 12]. A detailed description of Oncokompas can be found elsewhere [10, 12, 14, 16].

### Outcomes

The economic evaluation was conducted from a societal perspective, including direct medical costs (costs of healthcare resource use and medication), direct non-medical costs (traveling to and parking at healthcare services, costs of informal care, support groups), indirect non-medical costs (costs due to absence from paid work or loss of productivity from paid work), and intervention costs. All outcome measures were collected at baseline (time of inclusion), and at a 3-month and 6-month follow-up assessment. Since the follow-up of the study was 6 months, neither costs nor effects were discounted.

Direct medical and direct non-medical costs were measured with the Institute for Medical Technology Assessment (iMTA) Medical Consumption Questionnaire (iMCQ) [18]. The iMCQ measures the use of healthcare (e.g., number of visits to medical specialists, hospital admissions), other facilities (e.g., hours of informal care use, participation in support groups), and medication (e.g., painkillers, antihypertensive medication, endocrine therapy) in the past 3 months. Direct medical and direct non-medical costs were calculated as units of resource use multiplied by the integral cost price per unit [19, 20]. Direct non-medical costs of traveling to healthcare services were calculated as units of resource use multiplied by the average distance to the location, multiplied by the price per kilometre. All prices were adjusted to 2017 prices using the consumer price index.

Indirect non-medical costs were measured with the iMTA Productivity Costs Questionnaire (iPCQ) [21]. Productivity losses through absence from paid work (absenteeism) and through the reduced quality of performed paid work (presenteeism) were measured in the last 3 months. Productivity losses due to absenteeism were calculated as the number of days absent from work, and presenteeism as the number of days with less productivity multiplied by the estimated amount of lost quality of performed work on an 11-point scale. Absenteeism and presenteeism costs were calculated as productivity losses multiplied by the price of productivity costs per hour of paid work, using the friction cost approach for absenteeism, with a friction period of 85 days [20]. The price of one hour paid work was €36.38, irrespective of sex and age.

Health-related quality of life was measured with the EuroQol-5 Dimension (EQ-5D). The utility score was obtained using the Dutch index tariff [22].

Intervention costs of Oncokompas were calculated using a top-down approach. Costs for running Oncokompas (ICT, product and data management, content updating, implementation, and marketing) are estimated at €450,000 annually. When reaching 18,000 cancer survivors per year (16% of all newly diagnosed cancer patients in the Netherlands) [14, 23], the intervention costs are estimated at €25 per user.

### Statistical analysis

Analyses were performed using SPSS version 25 (IBM, Armonk, NY) and STATA version 14 (STATA, College Station, TX). Descriptive statistics, *χ*^2^ tests, and independent samples *t* tests were used to describe and compare baseline characteristics between the intervention and control group. To provide information on types of costs included in the analyses and their relative importance at each time point, data of complete cases (participants who completed baseline and both follow-up measurements) were used.

To test the cost-utility of Oncokompas compared with CAU, a base case intention-to-treat cost-utility analysis was performed, including all participants, with imputed data for missing time points, and estimated intervention costs of €25 per Oncokompas user. The robustness of this finding was tested by four additional sensitivity analyses in which the base case analysis:

1. Was adjusted for baseline EQ-5D scores and baseline total costs,
2. Included varying intervention costs of Oncokompas (range, €15 to €100 per user),
3. Was performed among survivors with complete data at all time-points,
4. Was performed from a healthcare perspective, including only direct medical costs and intervention costs.

In case data was missing on item level (e.g. a patient reported to have visited a general practitioner, but did not report the number of visits), assumptions were made based on means per allocation group and time point. In case data was missing on questionnaire level, missing data was imputed as total costs or utility score per time point per allocation group, using multiple imputations (predictive mean matching) by chained equations. Backward multivariable linear and logistic regression analyses were performed to investigate which variables (socio-demographic, clinical, and psychosocial variables at baseline) were associated with missing data, total costs, or utility scores. A description of these variables is listed in the Appendix. Variables that were found to be associated with missing data (EORTC QLQ-C30 summary score [24]), total costs (age, comorbidities, time since diagnosis, EORTC QLQ-C30 summary score), and utility scores (age, comorbidities, marital status, tumour stage, positive adjustment (subscale of the Mental Adjustment to Cancer (MAC) scale [25]), and employment status), and variables that differed statistically significant between intervention and control group at baseline (positive adjustment (MAC)) were included in the multiple imputation model. Ten imputed datasets were created and analysed separately, and the results of the 10 analyses were pooled, using Rubin’s (1987) rules.

The total cumulative costs per patient were calculated by summing costs measured with the iMCQ and iPCQ at 3- and 6-month follow-up and intervention costs in the intervention group. Quality-adjusted life-years (QALYs) were calculated as the EQ-5D utility scores per time point, multiplied by the corresponding time period (i.e. 3 months).

To obtain costs per QALY gained, an incremental cost-utility ratio (ICUR) was calculated as the incremental costs divided by incremental effects, with the following formula: (mean costs_intervention_ – mean costs_control_) / (mean QALYs_intervention_ – mean QALYs_control_). Uncertainty around the ICUR was assessed using bootstrapping with 5000 replications and was projected on a cost-utility plane.

## Results

**Fig. 1:**
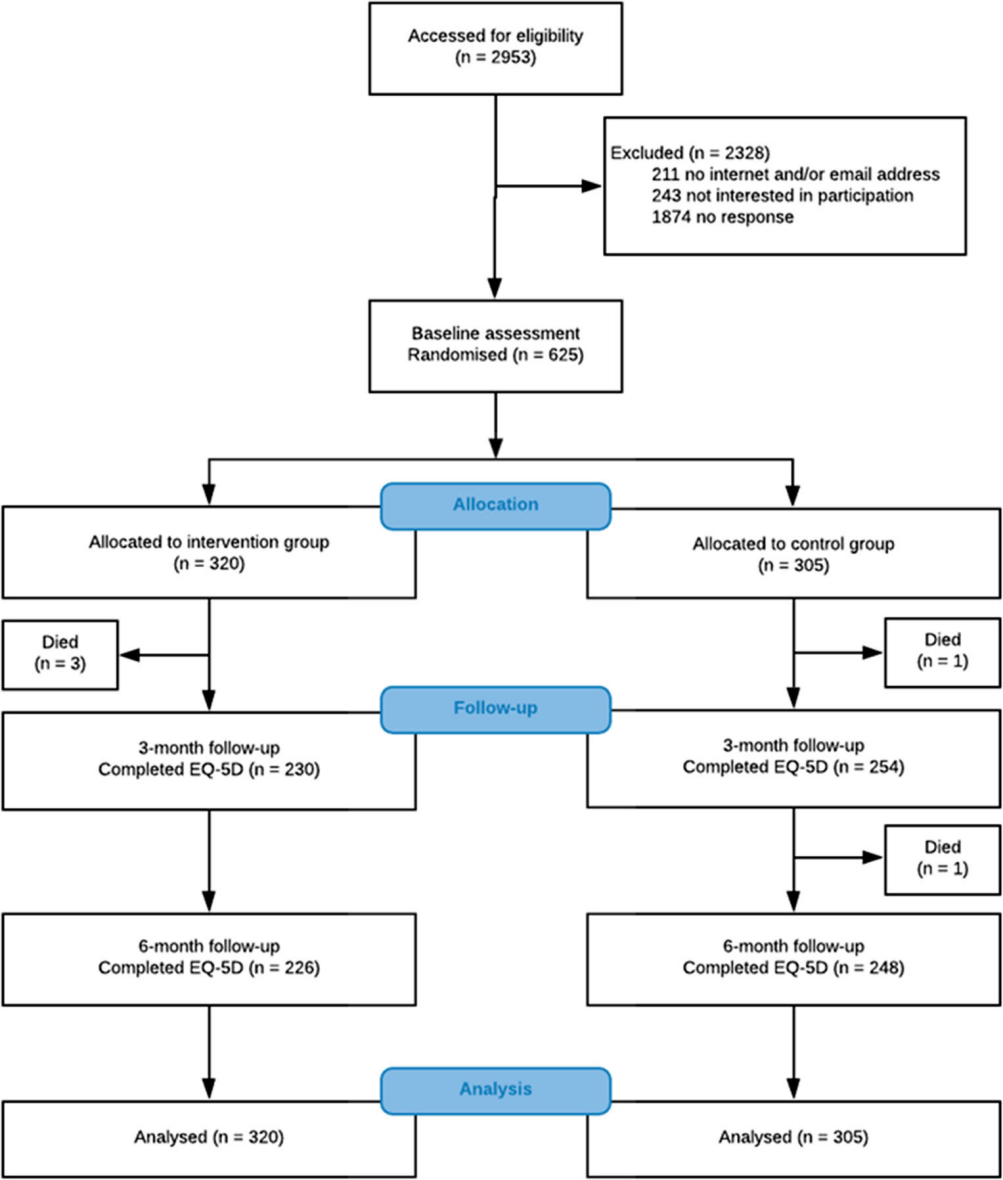
CONSORT flow diagram

In 14 participating hospitals throughout the Netherlands, 2953 cancer survivors were invited to participate between October 12, 2016, and May 24, 2018. In total, 625 (21%) survivors consented to participate, completed the baseline questionnaire at study inclusion, and were randomly allocated to the intervention (*n* = 320) or control (*n* = 305) group. Of them, respectively 205 (64%) and 240 (79%) completed both follow-up questionnaires and were complete cases. Figure 1 shows the Consolidated Standard of Reporting Trials (CONSORT) diagram of the study inclusion. There were no statistically significant differences in baseline socio-demographic and clinical characteristics between the intervention and control groups (Table 1).

**Table 1:** Baseline characteristics

Characteristic	Intervention (*N* = 320)	Control (*N* = 305)
Age, years	65 (56–71)	65 (57–71)
Women	158 (49%)	158 (52%)
Men	162 (51%)	147 (48%)
Education level		
Low	111 (35%)	117 (39%)
Medium	105 (33%)	85 (28%)
High	103 (32%)	100 (33%)
Missing	1 (<1%)	3 (1%)
Marital status, partner	265 (83%)	269 (88%)
Employment status, employed	122 (38%)	99 (33%)
Tumour type		
Breast cancer	66 (21%)	72 (24%)
Colorectal cancer	80 (25%)	72 (24%)
Head and neck cancer	99 (31%)	86 (28%)
Lymphoma	75 (23%)	75 (25%)
Tumour stage		
Stage I	106 (35%)	104 (36%)
Stage II	73 (24%)	70 (24%)
Stage III	61 (20%)	67 (23%)
Stage IV	64 (21%)	52 (18%)
Missing	16 (5%)	12 (4%)
Treatment		
None or single treatment	137 (43%)	124 (41%)
Multimodal treatment	183 (57%)	181 (59%)
Comorbidities		
*None or one comorbidity*	249 (78%)	229 (75%)
*Multiple comorbidities*	71 (22%)	76 (25%)
Time since diagnosis, months	25.0 (16–41)	29.0 (17–41)
*3–<12 months*	39 (12%)	38 (13%)
*12–<24 months*	104 (33%)	85 (28%)
*24–60 months*	177 (55%)	182 (60%)
Treatment (*n*, %)		
None or single treatment	137 (43%)	124 (41%)
Multimodal treatment	183 (57%)	181 (59%)
EORTC QLQ-C30 summary score	85.3 (14.9)	85.4 (13.6)

Data are mean (SD), *n* (%), or median (IQR)

The mean EQ-5D score of survivors at baseline was 0.89 (sd 0.15) in the intervention group and 0.87 (sd 0.17) in the control group (*p* = 0.11). The mean total costs in the previous 3 months at baseline in the intervention group were €1013 (sd 1760), compared with €1158 (sd 1936) in the control group (*p* = 0.33). The mean costs for survivors with complete data per time point per group are presented in Supplementary Table 1.

### Cost-utility analyses

**Fig. 2:**
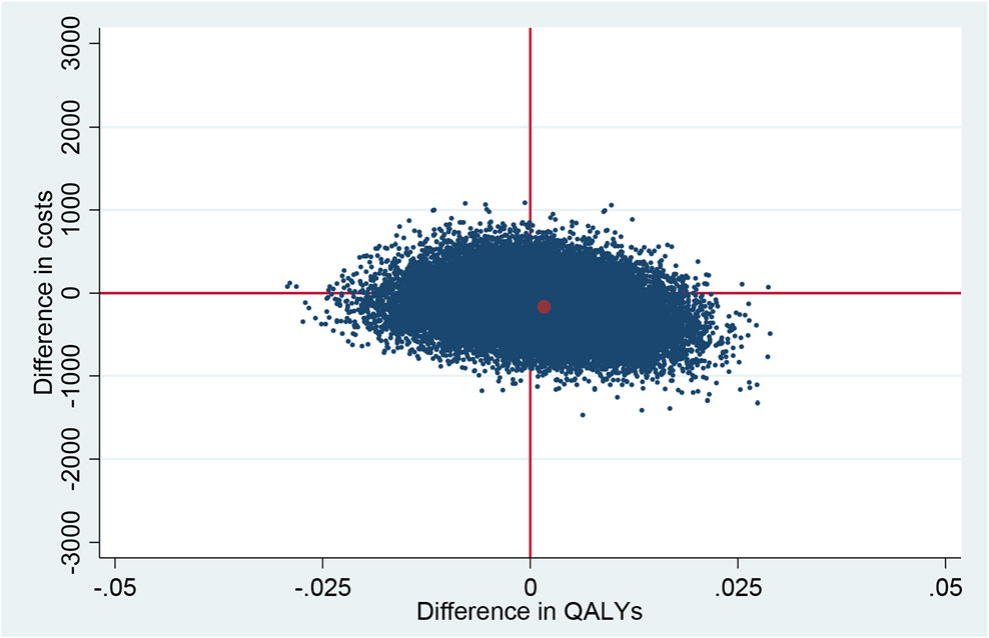
Cost-utility plane of the base case analysis. QALYs, quality-adjusted life years; NE, north-east; SE, south-east; SW, south-west; NW, north-west quadrant. The percentages indicate the percentage of bootstrap replications in a certain quadrant

The results of the cost-utility analyses are shown in Table 2 and Fig. 2. In the base case analysis, QALYs gained were similar in the intervention group compared with those in the control group (incremental effects, 0.0017; 95% CI, −0.0121 to 0.0155). The mean total costs in the intervention group were slightly, but non-significantly, lower than the mean total costs in the control group (incremental costs, − €163; 95% CI, − €665 to €326). Of the bootstrapped cost-utility pairs, 47% fell into the southeast quadrant, indicating that Oncokompas was more effective and less costly compared with CAU. The probability that the cumulative QALYs were higher in the intervention group compared with those in the control group was 60%, and the probability that Oncokompas was less costly compared with CAU was 73% (Fig. 2). To assess the robustness of this finding, four additional sensitivity analyses were performed as shown in Table 2.

1. When the base case analysis was corrected for baseline EQ-5D utility scores and costs, the probability that in the intervention group QALYs were higher was 13% (incremental effect, − 0.0057), and the probability that costs were lower was 51% (incremental costs, €2), compared with the control group. Because of these results, the subsequent sensitivity analyses were also corrected for baseline EQ-5D and costs.
2. Analyses with intervention costs of €15 and €100 showed that the probability that in the intervention group the QALYs were higher was 13% (incremental effects, − 0.0057), and the probability that costs were lower were 52% and 39% (incremental costs, − €8 and €77) respectively, compared with the control group.
3. Analyses on the complete cases showed that the probability that in the intervention group QALYs were higher was 30% (incremental effect, − 0.0023), and the probability that costs were lower was 41% (incremental costs, €68), compared with the control group.
4. Analyses with only direct medical costs taken into account showed that the probability that in the intervention group the QALYs were higher was 20% (incremental effect, − 0.0043), and the probability that costs were lower was 57% (incremental costs, − €40), compared with the control group.

**Table 2:** Results of the cost-utility analyses and base case and sensitivity analyses

Analysis		QALYs		Costs (€)		Incremental effects		Incremental costs	
Group	N	Mean	SEM	Mean	SEM	QALY	95% CI	€	95% CI
Base case						0.0017	− 0.0121 to 0.0155	− 163	− 665 to 326
Intervention	320	0.4452	0.0052	1935	224				
Control	305	0.4435	0.0045	2098	191				
Sensitivity analyses^1^									
Base case with correction for baseline						− 0.0057	− 0.0161 to 0.0048	2	− 441 to 443
Intervention	320	NA	NA	NA	NA				
Control	305	NA	NA	NA	NA				
Intervention costs									
*€15*						− 0.0057	− 0.0161 to 0.0048	− 8	− 451 to 433
Intervention	320	NA	NA	NA	NA				
Control	305	NA	NA	NA	NA				
*€100*						−0.0057	−0.0161 to 0.0048	77	− 366 to 518
Intervention	320	NA	NA	NA	NA				
Control	305	NA	NA	NA	NA				
Complete cases						− 0.0023	− 0.0112 to 0.0054	68	− 452 to 602
Intervention	205	NA	NA	NA	NA				
Control	240	NA	NA	NA	NA				
Healthcare perspective (direct medical costs)						− 0.0043	− 0.0148 to 0.0061	− 40	− 344 to 241
Intervention	320	NA	NA	NA	NA				
Control	305	NA	NA	NA	NA				

^1^All sensitivity analyses were corrected for baseline costs and EQ-5D utility score

## Discussion

This study investigated the cost-utility of a fully automated BIT ‘Oncokompas’ among cancer survivors, compared with CAU. In the base case analysis, QALYs were similar and costs were non-significantly lower in the intervention group (− €163), compared with those in the control group. When the base case analysis was corrected for baseline EQ-5D utility scores and costs, QALYs were non-significantly lower in the intervention group and costs were similar compared with those in the control group.

The finding that Oncokompas is more or less equally effective in terms of utilities and costs as CAU confirms our earlier research on the efficacy of Oncokompas [14]. That study showed that Oncokompas has a small positive effect on HRQOL, when measured with a cancer-generic questionnaire (EORTC QLQ-C30). However, that study also showed that the effects of Oncokompas were also found on tumour-specific symptoms (measured with EORTC tumour-specific modules). In the present study, QALYs are based on the generic EQ-5D, which does not take cancer-generic and tumour-specific symptoms into account. Also, ceiling effects were found in the EQ-5D utility scores, as many participants had a high or the maximum EQ-5D score at baseline, and the group performed relatively well at baseline on other outcome measures. In future research, the EORTC QLU-C10D can be used for measuring utilities, of which data can be derived from the QLQ-C30 [26].

The previous study also showed that for some tumour-specific symptoms, beneficial effects of Oncokompas occurred at 3- or even 6-month follow-up, and not directly post-intervention [14]. It may be that survivors need time to follow-up on the provided advice and use the preferred interventions or supportive care options, before seeing improvements in HRQOL. The possible subsequent cost-saving effect as a result of the improvements is expected to take even longer to become visible. Since the follow-up period of the study was only 6 months, it is possible that this long-term cost-saving effect was not captured within the follow-up period in this study. More research is therefore needed on long-term effects of BITs such as Oncokompas, for instance by performing a budget impact analysis on real-world data, when Oncokompas has been implemented and scaled up in clinical practice. Together with the results on the efficacy of Oncokompas [14] and the fact that Internet skills of cancer survivors are expected to improve over time and eHealth will be more commonly used, it is expected that long-term cost-utility of BITs such as Oncokompas will be even more positive. It is expected that the effects related to the use of the proposed healthcare options in Oncokompas will positively influence utilities (i.e. better quality of life and higher QALYs), as well as reduce costs (as a result of (earlier) use of healthcare options or applying self-help advice, more expensive treatment and productivity losses can be prevented or reduced). Performing these analyses on real-world data, it is possible to measure over a longer period in time and include all survivors, which improves the generalizability of the results. Furthermore, implementation and upscaling will lead to more users, which leads to less intervention costs per user, which improves the cost-utility in favour of Oncokompas.

With an increasing number of cancer survivors, the costs of cancer survivorship healthcare are growing, together with an increasing healthcare workforce shortage [1, 27]. The present study showed that from a healthcare perspective (taking only direct medical costs into account), costs were not significantly lower (− €40) in the intervention group. It is promising that the tailored approach in Oncokompas does not seem to lead to increased medical costs: users are encouraged to apply personalized tips and information provided within Oncokompas and use self-help interventions first, before turning to professional care. This may prevent worsening of symptoms and may be cost-saving in the long-term.

Economic evaluations of eHealth interventions among patients with chronic diseases are scarce and mostly performed among patients with diabetes and cardiovascular disease [28, 29]. To the best of our knowledge, this study was the first economic evaluation of an eHealth intervention among cancer survivors. A strength of this study is that we performed the cost-utility analysis from a societal as well as a healthcare perspective.

Potential limitations of this study were that several assumptions were made regarding missing data on healthcare resource use. Missing data was replaced based on assumptions, or imputed using multiple imputation techniques. This might not reflect reality, but since we made similar assumptions and imputations in both groups, it is not expected that this has influenced our findings. Also, since the cost prizes of unit resource and productivity costs in this study are based on the Dutch tariff, the results might not be generalizable to other countries. The Dutch healthcare system and reimbursement of care, and thereby the low barrier to seek care, might also not be representative of other countries. Furthermore, the attrition rate was higher in the intervention group than in the control group, and there were also more complete cases in the control group than in the intervention group. This might be explained by the fact that participants in the wait-list control group obtain access to Oncokompas after the last follow-up measurement, which might have been an extra motivation to complete the follow-up assessments. We cannot be sure whether this has under- or overestimated the results, but since the results from the sensitivity analysis with only taken into account the complete cases did not differ much from the sensitivity analysis with all participants, it is expected that this influence is limited. Finally, the participation rate of the randomized controlled trial was 21%, and participants were mostly long-term survivors and had relatively good baseline scores, which might limit the generalizability of the results. Further research is needed to see whether these results can be confirmed among representative samples of cancer survivors, also diagnosed with other tumour types.

In conclusion, results indicate that a fully automated BIT such as Oncokompas is at least as effective as usual cancer survivorship care, and not more expensive. Implementation and upscaling of Oncokompas may help to improve cancer survivorship care in a (cost-)effective manner.

### Electronic supplementary material

ESM 1 (DOCX 23 kb).

## Appendix

### Socio-demographic and clinical characteristics

Socio-demographic and clinical characteristics were measured with a study-specific questionnaire (marital status, employment status, comorbidities), or extracted from the Netherlands Cancer Registry (NCR) (age, tumour stage, time since cancer diagnosis).

### Health-related quality of life

The summary score (SumSC) of the EORTC QLQ-C30 is based on the five functional scales (physical, cognitive, emotional, social, and role functioning), three symptom scales (fatigue and nausea/vomiting, and pain), and five single items (dyspnoea, insomnia, appetite loss, constipation, diarrhoea) of the QLQC30. The SumSC ranges from 0 to 100. A higher SumSC score represents better HRQOL [24].

### Mental adjustment to cancer

The Mental Adjustment to Cancer (MAC) scale comprises two summary subscales: summary positive adjustment (SPA) and summary negative adjustment (SNA). Scores range from 17 to 68 on SPA, with a higher score indicating more positive adjustment, and 16 to 64 on SNA, a higher score indicating more negative adjustment [25].
