# Supplementary material for: Cost-utility of an eHealth application ‘Oncokompas’ that supports cancer survivors in self-management: results of a randomised controlled trial
Source: J Cancer Surviv. 2020 Jul 12;15(1):77–86. doi: 10.1007/s11764-020-00912-9 (PMC7822793; doi:10.1007/s11764-020-00912-9)
Supplement: Supplementary file 1 — (DOCX 23 kb). [file 11764_2020_912_MOESM1_ESM.docx]

**Supplementary Table 1** - Frequencies, mean costs (sd) (€) per time point for complete cases

|  | |  | **Baseline (T0)** | | | | | | **3-months follow-up (T2)** | | | | | | **6-months follow-up (T3)** | | | | | |
| --- | --- | --- | --- | --- | --- | --- | --- | --- | --- | --- | --- | --- | --- | --- | --- | --- | --- | --- | --- | --- |
|  | |  | Intervention  (*N* = 205) | | | Control  (*N* = 240) | | | Intervention  (*N* = 205) | | | Control  (*N* = 240) | | | Intervention  (*N* = 205) | | | Control  (*N* = 240) | | |
|  | | Price* | % | Mean | SD | % | Mean | SD | % | Mean | SD | % | Mean | SD | % | Mean | SD | % | Mean | SD |
| **DIRECT MEDICAL COSTS** | | |  | **639** | **1,215** |  | **816** | **1,540** |  | **510** | **803** |  | **625** | **1,135** |  | **600** | **1,407** |  | **625** | **1,688** |
| General practitioner | |  |  |  |  |  |  |  |  |  |  |  |  |  |  |  |  |  |  |  |
|  | *Phone* | 17 | 39% | 11 | 17 | 32% | 9 | 17 | 34% | 9 | 26 | 34% | 10 | 18 | 26% | 7 | 14 | 38% | 10 | 17 |
|  | *Home visit* | 51 | 2% | 2 | 18 | 4% | 4 | 26 | 1% | 0 | 4 | 3% | 2 | 19 | 1% | 0 | 5 | 3% | 2 | 14 |
|  | *Consultation* | 34 | 53% | 31 | 43 | 49% | 31 | 44 | 46% | 27 | 37 | 49% | 33 | 69 | 40% | 27 | 47 | 43% | 30 | 49 |
|  | *Nurse consultation* | 17 | 2% | 0 | 5 | 0% | 0 | 4 | 2% | 1 | 6 | 1% | 0 | 4 | 0% | - | - | 1% | 0 | 3 |
| Company doctor | | 70 | 7% | 5 | 21 | 6% | 6 | 27 | 4% | 3 | 16 | 6% | 6 | 25 | 4% | 3 | 18 | 5% | 5 | 24 |
| Social worker | | 67 | 3% | 4 | 26 | 3% | 3 | 15 | 2% | 4 | 36 | 3% | 4 | 28 | 3% | 5 | 27 | 3% | 4 | 28 |
| Physiotherapist | | 34 | 24% | 74 | 187 | 36% | 134 | 268 | 27% | 81 | 182 | 34% | 127 | 255 | 27% | 70 | 170 | 30% | 95 | 206 |
| Ergotherapist | | 34 | 3% | 3 | 25 | 2% | 2 | 17 | 1% | 0 | 3 | 0% | - | - | 1% | 0 | 2 | 0% | 0 | 2 |
| Dietitian | | 30 | 6% | 3 | 14 | 9% | 4 | 16 | 5% | 2 | 11 | 7% | 6 | 31 | 6% | 2 | 11 | 6% | 4 | 15 |
| Speech therapist | | 31 | 2% | 2 | 26 | 2% | 2 | 17 | 2% | 3 | 30 | 3% | 3 | 37 | 1% | 3 | 43 | 2% | 2 | 14 |
| Oral hygienist | | 26 | 25% | 7 | 14 | 24% | 7 | 15 | 23% | 6 | 12 | 23% | 7 | 15 | 22% | 6 | 12 | 27% | 8 | 18 |
| Psychologist/psychiatrist | | 96-126 | 4% | 17 | 115 | 6% | 17 | 84 | 3% | 19 | 135 | 4% | 13 | 91 | 6% | 15 | 71 | 5% | 21 | 144 |
| Medical specialist | |  |  |  |  |  |  |  |  |  |  |  |  |  |  |  |  |  |  |  |
|  | *General hospital* | 82 | 55% | 99 | 181 | 60% | 95 | 123 | 43% | 69 | 101 | 46% | 80 | 185 | 46% | 71 | 113 | 46% | 77 | 157 |
|  | *Academic hospital* | 167 | 36% | 99 | 217 | 30% | 93 | 264 | 31% | 72 | 138 | 23% | 63 | 161 | 25% | 70 | 149 | 23% | 50 | 108 |
| Spiritual counsellor | | 132 | 2% | 5 | 41 | 1% | 4 | 37 | 1% | 1 | 9 | 2% | 5 | 46 | 2% | 2 | 16 | 3% | 6 | 40 |
| Alternative treatment | | 62 | 4% | 11 | 69 | 5% | 10 | 50 | 8% | 9 | 38 | 7% | 22 | 140 | 6% | 10 | 58 | 4% | 9 | 47 |
| Emergency care visit | | 265 | 4% | 16 | 77 | 7% | 23 | 89 | 3% | 8 | 45 | 4% | 11 | 53 | 3% | 12 | 66 | 4% | 10 | 50 |
| Ambulance to hospital | | 527 | 2% | 10 | 73 | 3% | 13 | 82 | 1% | 3 | 37 | 1% | 4 | 48 | 2% | 8 | 63 | 1% | 4 | 48 |
| Day treatment | |  |  |  |  |  |  |  |  |  |  |  |  |  |  |  |  |  |  |  |
|  | *Hospital* | 310 | 7% | 77 | 489 | 6% | 81 | 579 | 7% | 65 | 288 | 4% | 22 | 123 | 9% | 82 | 561 | 5% | 56 | 433 |
|  | *Care center^***^* | 69-313 | 1% | 3 | 44 | 0% | 29 | 444 | 1% | 3 | 48 | 0% | - | - | 0% | - | - | 0% | - | - |
| Admission | |  |  |  |  |  |  |  |  |  |  |  |  |  |  |  |  |  |  |  |
|  | *Hospital* | 487 | 4% | 105 | 635 | 7% | 162 | 765 | 5% | 86 | 547 | 5% | 112 | 734 | 7% | 121 | 649 | 4% | 73 | 661 |
|  | *Care center^***^* | 172-471 | 0% | - | - | 0% | - | - | 0% | - | - | 0% | 13 | 199 | 1% | 39 | 559 | 0% | 75 | 1,157 |
| Personal care | | 51 | 1% | 0 | 7 | 0% | 3 | 43 | 0% | - | - | 1% | 5 | 58 | 0% | - | - | 1% | 11 | 138 |
| Nursing care | | 75 | 1% | 3 | 38 | 2% | 17 | 194 | 0% | - | - | 1% | 13 | 189 | 0% | - | - | 1% | 13 | 189 |
| Medication | | 0-38 | 66% | 51 | 163 | 70% | 65 | 206 | 66% | 39 | 66 | 69% | 63 | 188 | 65% | 46 | 162 | 73% | 58 | 183 |
| **DIRECT NON-MEDICAL COSTS** | | |  | **204** | **782** |  | **235** | **597** |  | **139** | **490** |  | **252** | **857** |  | **146** | **434** |  | **227** | **609** |
| Transport *^****^* | | 0-9 | - | 18 | 26 | - | 19 | 27 | - | 14 | 18 | - | 16 | 23 | - | 14 | 19 | - | 15 | 26 |
| Home care | | 20 | 2% | 5 | 44 | 3% | 16 | 123 | 2% | 8 | 68 | 2% | 5 | 48 | 2% | 10 | 73 | 2% | 9 | 91 |
| Informal care | | 15 | 10% | 117 | 714 | 12% | 80 | 338 | 11% | 71 | 376 | 14% | 118 | 747 | 13% | 79 | 373 | 12% | 75 | 374 |
| Supportive care*^*****^* | | 15-65 | 8% | 64 | 276 | 13% | 120 | 427 | 7% | 46 | 242 | 10% | 113 | 426 | 6% | 43 | 208 | 12% | 128 | 468 |
| **INDIRECT NON-MEDICAL COSTS** | | | | **124** | **708** |  | **160** | **837** |  | **193** | **1,397** |  | **251** | **1,385** |  | **240** | **1,526** |  | **95** | **518** |
| Absenteeism | | 36/hour | 7% | 114 | 705 | 11% | 147 | 814 | 5% | 187 | 1,378 | 9% | 241 | 1,369 | 5% | 235 | 1,523 | 7% | 88 | 507 |
| Presenteeism | | 36/hour | 10% | 9 | 46 | 11% | 14 | 92 | 6% | 6 | 37 | 10% | 10 | 43 | 8% | 4 | 21 | 10% | 7 | 36 |
| **TOTAL COSTS** | |  |  | **967** | **1,778** |  | **1,211** | **2,021** |  | **842** | **1,711** |  | **1,128** | **2,058** |  | **986** | **2,221** |  | **947** | **2,002** |

* Reference price per unit (€) ** Psychologic or psychiatric help = psychological help at a private practice (€96), mental health service (out-patient) (€100), addiction clinic (€126), and/or psychologic help in hospital (€126) *** Care center = residential center (treatment: €69, admission: €172), rehabilitation center (treatment: €313, admission: €471) and/or psychiatric institution (treatment: €173, admission: €309); **** Transport = transportation and parking costs: €0.21/km + €3.07 parking costs per visit; ***** Self-management interventions = help with coping (€65), support groups (€65), sport rehabilitation programs (€65), body image care (€15) and/or online self-help programs (€15) %: Percentage of participants that used the care at least once
